# Variation in the resource environment affects patterns of seasonal adaptation at phenotypic and genomic levels in *Drosophila melanogaster*

**DOI:** 10.1093/evlett/qraf031

**Published:** 2025-09-22

**Authors:** Jack K Beltz, Mark Christopher Bitter, August Goldfischer, Dmitri A Petrov, Paul Schmidt

**Affiliations:** Department of Biology, University of Pennsylvania, Philadelphia, Pennslyvania, United States; Department of Biology, Stanford University, Stanford, California, United States; Department of Biology, University of Pennsylvania, Philadelphia, Pennslyvania, United States; Department of Biology, Stanford University, Stanford, California, United States; Department of Biology, University of Pennsylvania, Philadelphia, Pennslyvania, United States

**Keywords:** adaptation, seasonality, environmental heterogeneity, phenotypic evolution, resource variation, genomic evolution

## Abstract

Natural populations often experience heterogeneity in the quality and abundance of environmentally acquired resources across both space and time, and this variation can influence population demographics and evolutionary dynamics. In this study, we directly manipulated diet in replicate populations of *Drosophila melanogaster* cultured in experimental mesocosms in the field. We found no significant effect of resource variation on estimates of adult census size. Resource variation altered patterns of phenotypic and genomic evolution across replicate populations; however, we find that this effect is secondary to selection driven by the fluctuating seasonal environment. Seasonal adaptation was observed for all traits assayed and elicited genome-wide signatures of selection. In contrast, adaptation to the resource environment was trait-specific and exhibited an oligogenic architecture. This illustrates the capacity of populations to adapt to a specific axis of variation (the resource environment) without hindering the adaptive response to seasonal change. This, in turn, suggests that resource variation may be an important force driving fluctuating selection across natural populations, ultimately contributing to the maintenance of genetic and phenotypic variation.

## Introduction

Identifying the environmental factors that shape population demographics and evolutionary dynamics remains a fundamental goal in ecology and evolution. A major component of variation in natural habitats is the availability and quality of resources (e.g., water, light, nitrogen, food supply, etc.). Variation in the quality or availability of critical resources can impact the growth and productivity of populations (Blaustein et al., 2003; Gilg et al., 2003; Schindler, 1974) and alter selection pressures that shift patterns of adaptive evolution (e.g., Grant & Grant, 1989). As ecological and evolutionary processes can occur on the same timescales (Hendry & Kinnison, 1999; Hendry et al., 2007; Johnston & Selander, 1964; Reznick et al., 1997), variation in the quality and availability of environmental resources may simultaneously impact both ecological (e.g., population size) and evolutionary (e.g., trait value) outcomes of populations.

Environmental quality affects population demographics in various systems and contexts (Belovsky et al., 1999; Franken & Hik, 2004; Griffen & Drake, 2008; Hakoyama et al., 2000; Klok & De Roos, 1998; MacArthur, 1984; Philippi et al., 1987; Pimm et al., 1988). Furthermore, the quality and availability of environmentally acquired resources are used as predictors when estimating the growth rate and carrying capacity of populations (Hobbs et al., 1982) and may also affect different components of fitness (Chatelain et al., 2021; Mitrovski & Hoffmann, 2001). Resource-limited population growth is especially prevalent in systems where water or food supplies are seasonal or ephemeral, impacting local rates of extinction (Longman et al., 2023; Schindler, 1974).

Adaptive responses to variation in the biotic and abiotic environment can occur over extremely short intervals (Bitter et al., 2024; Grainger et al., 2021; Rudman et al., 2022), reinforcing the notion that evolutionary and ecological processes can occur on the same timescale and potentially interact (Carroll et al., 2007; Hairston et al., 2005). One of the mechanisms by which populations change in response to environmental variation is adaptive tracking (Bitter et al., 2024; Rudman et al., 2022). In environments where resources are variable, and this variability creates distinct selection pressures, populations could potentially track shifts in resource abundance and/or composition. What remains unknown is whether populations can adaptively track the distinct selective regimes associated with the fine-scale variation in environmental resources that shift over short timescales (e.g., weeks to months, 2–5 generations). Additionally, if this is the case, the extent to which such resource variation simultaneously impacts both ecological and evolutionary dynamics in natural populations remains unresolved. Alternatively, variation in the resource environment may be associated with other evolutionary responses to environmental heterogeneity, such as adaptive plasticity (Brooker et al., 2022; Charmantier et al., 2008; Ghalambor et al., 2007) or bet-hedging (Gillespie, 1977; Simons, 2011; Stearns, 1976).

Natural populations of *Drosophila melanogaster* experience pronounced seasonal variation in the resource environment: In their ancestral range in sub-Saharan Africa, the putative ancestral substrate, marula (Mansourian et al., 2018) is seasonally ephemeral, as are the predominant fruit substrates in temperate regions (Markow, 2015; Reaume & Sokolowski, 2006). Seasonal environmental change, including variation in resource availability, affects population size (Dempster et al., 1995; Lawson et al., 2015), relative abundance of competing *Drosophila* species (Grainger et al., 2021), and patterns of rapid adaptation at both the phenotypic (Behrman & Schmidt, 2022; Behrman et al., 2015; Schmidt & Conde, 2006) and genomic (Bergland et al., 2014; Machado et al., 2021) levels. Similarly, experiments conducted in field mesocosms over the spring-to-fall seasonal progression have shown that *D. melanogaster* populations adaptively track shifts in the biotic and abiotic environment over weekly to monthly timescales (Bitter et al., 2024; Grainger et al., 2021; Rudman et al., 2019, 2022). However, the role of variation in the resource environment in contributing to the ecological and evolutionary dynamics of populations remains unresolved.

Dietary manipulations in the laboratory have revealed the direct influence of caloric and macronutrient variation on *D. melanogaster* life history traits, including viability, fecundity, body size, lifespan, and appendage size (Bakker, 1959, 1962; Chiang & Hodson, 1950; Rodrigues et al., 2015; Shingleton et al., 2017; Skorupa et al., 2008; Wang & Clark, 1995). Diet variation also affects patterns of genomic evolution (Kawecki et al., 2021) and multiple phenotypes, including mate choice (Schultzhaus et al., 2017) and ethanol tolerance (Cavener & Clegg, 1981). Natural populations harbor genotype-dependent variation for macronutrient tolerance (Havula et al., 2022), suggesting populations can evolve in response to shifts in resource availability.

Here, we conducted a manipulative field experiment to examine the effects of diet on population dynamics and patterns of seasonal adaptation. Throughout a 5-month experiment, we tracked census size bi-weekly, as well as fitness-associated phenotypes and genome-wide allele frequencies at five timepoints across replicate populations to determine how manipulation of the nutritional environment alters ecological dynamics, phenotypic trajectories, and patterns of genomic evolution. We hypothesized that the dietary treatment would affect population size as well as the selective landscape, resulting in divergent patterns of adaptation over seasonal time.

## Methods

### Field mesocosm experimental setup

Experimental populations were created using the same methodology as described in Rudman et al. (2022). Briefly, a panel of 80 inbred lines originally collected in June 2012 (Linvilla Orchards, Media, PA, USA) was outcrossed and expanded for four generations of recombination. In the next generation, experimental flies were collected as a single 12 hr cohort from density-controlled cultures; collections were combined in groups of 2,500 flies (equal sex ratios) and randomly assigned to an experimental treatment and replicate field cage (*N* = 6 cages per treatment). Additional details are provided in the supplemental methods section.

Two distinct food substrates were developed to simulate unique resource environments. Our low-quality (LQ) nutritional environment is largely pureed apples and mimics a natural diet regularly experienced in temperate *D. melanogaster* populations. Alternatively, the high-quality (HQ) nutritional environment is a standard cornmeal molasses-based medium used in laboratory culture and prior experiments in this system (Bitter et al., 2024; Rajpurohit et al., 2017; Rudman et al., 2019). The principal differences between the nutritional treatments are caloric content and the ratio of protein to carbohydrates (recipes and nutritional information given in Table S1).

Each population was maintained outdoors in an 8 m^3^ mesh enclosure containing a dwarf peach tree at the Pennovation Center of the University of Pennsylvania in Philadelphia, PA. The only food source and egg-laying substrate was 400 ml of the respective dietary treatment contained in 900 ml aluminum loaf pans, which were replaced every other day for the duration of the experiment (July 11–November 23, 2020). After 2 days of oviposition, each loaf pan was covered with a screen mesh lid, and embryos were allowed to develop separately in a small cage (30 cm × 30 cm × 30 cm) until eclosion ceased. The small cages were housed on the bottom of the shelving unit that holds the food supply, sheltered from wind, rain, and direct sunlight; further details and descriptions are given in the supplemental methods. A visual diagram of the mesocosm design and sampling methods is given in Figure S1.

### Measurement of population size and fitness-associated phenotypes

Population size was estimated bi-weekly to observe how the nutritional treatments affected population dynamics. Thirty minutes prior to sunset, four 0.5 m^2^ permanent ceiling quadrats (each representing 2.5% of the total cage surface area) in each cage were photographed to subsample the total adult populations across all mesocosms. The number of adult *D. melanogaster* in each sample photograph was counted (using a modified cell counting protocol with ImageJ [O'Brien et al., 2016]) and corrected for the total mesocosm surface area to estimate population size (Rudman et al., 2019, 2022).

In addition to population demographics, we phenotyped each population for a variety of fitness-associated traits that respond to variability in the seasonal and resource environments (Behrman & Schmidt, 2022; Behrman et al., 2015; Rudman et al., 2022). Our primary goal was to examine changes in the genetic composition of experimental populations over seasonal time and whether patterns of evolutionary change were altered by dietary treatment. Thus, all phenotypes at all timepoints were assayed in a common garden, and laboratory environment. Eggs were collected overnight from each population on fresh loaf pans and returned to the lab for two generations of density-controlled (30 ± 5 individuals, contained in narrow vials [Fly Stuff 32–116]) culture at 25 °C, 12L:12D on their respective diets. Fitness-associated phenotypes of the F2 generation were measured for each field cage on density and age-controlled replicates (*N* = 3 per cage per timepoint). Fecundity was measured as the total number of eggs laid by a group of five females over 3 days (Paaby et al., 2014). The development time for males and females was estimated as time from oviposition to eclosion for groups of 50 eggs by recording eclosion events 3× per day at 09:00, 13:00, and 17:00 and scoring by sex (Behrman et al., 2015); viability was estimated as the percentage of eggs laid that emerged as adults. Starvation resistance was measured separately for males and females as the time to death for replicate groups of 10 individuals kept in vials on 1% agar. Thorax length (a proxy for body size) was measured as the longest length across the dorsal shield in lateral view for 15 ethanol-preserved females. Measurements were recorded using a Leica MZ9.5 microscope, with an Olympus DP73 camera and CellSens standard measuring software (Betancourt et al., 2021). We assayed each of these phenotypes in the founding population as well as all experimental populations at five timepoints over the experiment; additional details are provided in the supplemental materials and methods. All project data and analyses are available at https://github.com/jkbeltz/EVL3-25-0011.R1.git and https://doi.org/10.5061/dryad.t1g1jwtfr.

Temperature data were measured using a shielded, non-aspirated temperature/relative-humidity sensor (CS-215L) mounted at 2 m height, located centrally among populations, and logged with a CR-1000 datalogger (all Campbell Scientific, Logan, UT). Temperature was logged every 15 min as an average of the previous 5 min. These data were used to construct degree day models (Kamiyama et al., 2020) to estimate generation times between each sampling interval throughout the experiment. 180-degree days were used to represent a single generation. Rates of phenotypic evolution were then calculated in haldanes (Gingerich, 2001) for all traits across the entire experiment as well as between each sampling interval.

In addition to the standard, common garden phenotyping of all cage populations at all timepoints on their diet of origin, an additional protocol was conducted at two timepoints (t2 on September 18, representing the end of the summer period and expansion, and t4 on October 25, before the winter collapse). Eggs were collected overnight from each population on fresh loaf pans on their respective diets and returned to the lab. After one generation of common garden culture, individuals from each population were then reared on both diets for the second generation (to control for maternal and carryover effects) to examine (1) diet-associated plasticity for the five assayed phenotypes and (2) whether such patterns of plasticity changed throughout the experiment. Fitness-associated phenotypes were measured on density- and age-controlled replicates in the F2 generation following an identical protocol as described above (further details given in supplemental methods).

Phenotypic data were analyzed in R (v4.3.2) using mixed-effect linear models, where time, mesocosm treatment, and/or phenotyping treatment were fixed variables, and individual cage was a random effect (lme4 [Bates, 2015], fit by REML; additional details provided in supplemental materials and methods). *T*-tests, analysis of variance (ANOVA) using the Satterthwaite method (Satterthwaite, 1946), and multivariate analysis of variance (MANOVA) were utilized to test the significance of phenotypic variation across treatments in multidimensional space.

### Whole genome sequencing and allele frequency estimation

**Figure 1.**
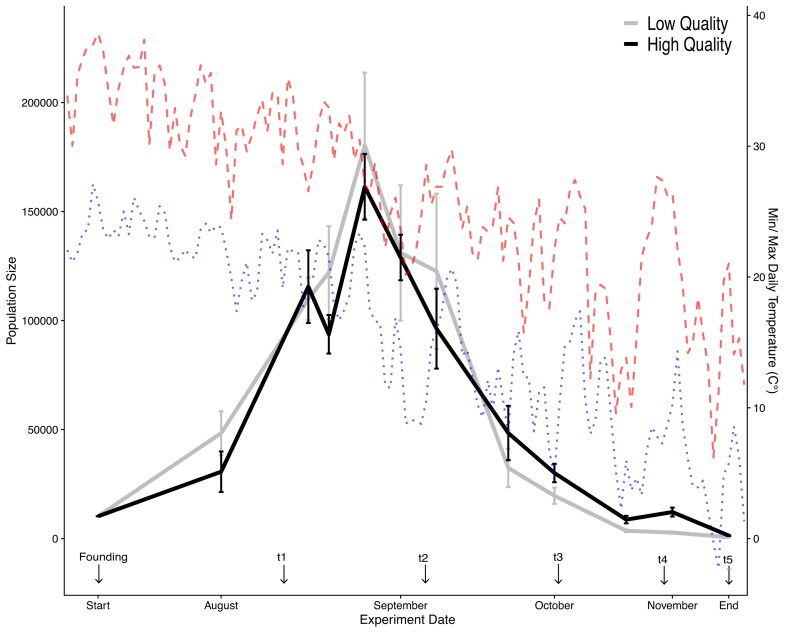
Mean (± *SE*) estimated adult census size across seasonally evolving populations of *Drosophila melanogaster* reared in either a low- or high-quality nutritional environment, along with the daily minimum (short-dash) and maximum (long-dash) temperatures throughout the experiment. Census estimates were obtained on 7/30, 8/12, 8/21, 8/30, 9/2, 9/18, 9/26, 10/1, 10/20, 11/2, and 11/14/20. The founding of the experimental populations and all phenotyping sampling timepoints are labeled on the *x*-axis.

We examined how the experimental manipulation affected patterns of allele frequency change over seasonal time. Specifically, during each of the five collection timepoints (Figure 1), 100 F1 adult female flies were randomly selected from each cohort of field-collected eggs, preserved in 80% ethanol, and stored at −80 °C. An additional four replicate samples were collected from the founding population used to initiate the experiment. Genomic DNA was later extracted from all pools using the New England Biolabs Monarch Genomic DNA Purification Kit. Whole-genome sequencing libraries were derived from each pooled sample with the Illumina DNA Prep Tagmentation kit, followed by sequencing on the Illumina NovaSeq 6000 flow cell using 150-bp, paired-end sequencing reads. Raw sequencing reads were trimmed of adapter sequences and bases with quality score <20 and subsequently aligned to the v5.39 *D. melanogaster* genome using bwa and default parameters (Li & Durbin, 2009). Deduplication of aligned reads was then conducted using Picard tools (http://broadinstitute.github.io/picard/). We downsampled aligned reads for each sample to obtain an equivalent, genome-wide coverage of 7× across samples. Haplotype-informed allele frequencies were then computed for each sample using the genome sequences of the founding inbred strains, a local inference method (Kessner et al., 2013), and a previously published pipeline (Tilk et al., 2019). This method produces an accuracy of allele frequency estimates at a genome-wide depth of 5× comparable to standard pooled sequencing approaches and 100× coverage (Rudman et al., 2022; Tilk et al., 2019). The final set of allele frequencies was then filtered to obtain only those single-nucleotide polymorphisms (SNPs) with an average minor allele frequency >0.02 in founder populations and >0.01 in at least one of the evolved samples, resulting in 1.9 M SNPs for our final assessment of genomic changes throughout the experiment. Raw sequencing reads associated with the allele frequency data analyzed in this experiment are publicly available as an NCBI SRA under the BioProject ID: PRJNA1306087.

### Analysis of genome-wide allele frequencies

Analysis of genome-wide allele frequency data was conducted in R v. 3.5.6. We first quantified and visualized patterns of genomic variation across all collected samples using principal component analysis (*prcomp* STATS package [R Core Team 2024]). Allele frequencies were centered and scaled prior to principal component analysis (PCA), and samples were projected onto the first, second, and third principal components. Through this, we interrogated whether variance in genome-wide allele frequencies throughout the experiment was predominantly driven by collection timepoint, treatment, or a combination thereof.

Next, we identified individual SNPs with systematic frequency movement across replicates throughout the experiment using a generalized linear model (GLM) where time was used as a continuous predictor. We initially ran this model independently for allele frequency data from each treatment. As drift is unlikely to produce coordinated shifts in allele frequencies across independent populations, we inferred that SNPs identified via this approach are subject to direct or linked selection in response to shared seasonal selection pressures. Prior to GLM regression, allele frequencies were weighted by effective coverage (Tilk et al., 2019) and the total number of chromosomes sequenced (*N* = 200). The normalized frequencies were fit to a GLM of the form allele frequency ∼ timepoint, using a quasi-binomial error variance structure (Wiberg et al., 2017). *p*-values associated with the coefficient of this GLM were adjusted using a Benjamini–Hochberg false discovery rate correction, and the distribution of significance values as a function of chromosomal position was then visualized using Manhattan plots.

Next, we quantified the extent to which patterns of selection were parallel between treatments. Specifically, we reciprocally quantified the behavior of the rising allele at SNPs in the identification treatment via GLM (false discovery rate [FDR] < 0.05 and allele frequency shift > 2%) iteratively and independently in each replicate of the reciprocal treatment from timepoint 1 to 5. We then compared each set of SNPs to a matched control set (matched on chromosomal arm and starting frequency) using a two-tailed paired *t*-test to ask whether allele frequency change between timepoints 1 and 5 exceeded background shifts at control sites and whether the dominant direction of movement was conserved in the test cage/treatment (FDR < 0.05). For visualization purposes, we plotted the median shift of the target and matched control SNPs for each iteration of this analysis, separately for each chromosomal arm and genome-wide.

We further quantified the extent to which patterns of genomic selection were parallel between treatments by computing Pearson correlation coefficients in allele frequency shifts across all timepoints between the HQ and LQ treatments. Correlations were computed genome-wide, as well as separately for SNPs on each chromosomal arm. The significance of these correlations was inferred using permutations of the allele frequency data across sites (*N* = 100 permutations per comparison). Next, to ask whether those loci putatively under selection within each treatment exhibited elevated, correlated movement between treatments (as would be expected if the parallelism between treatments was dominated by loci under selection identified independently within each treatment), we re-conducted this analysis using those SNPs with strong evidence of selection (or linked selection) identified via GLM independently for each treatment (FDR < 0.05 and allele frequency change > 2%; *N* = 29,303 SNPs identified in HQ and *N* = 2,203 sites in LQ). We then compared the resulting genome-wide and chromosomal-arm correlations for these sites to a distribution of correlations using random subsets (*N* = 100) of SNPs of the same number as our focal comparisons.

Non-independence (i.e., physical linkage) of SNPs is likely to induce correlated patterns of GLM significance across the genome, a dynamic not explicitly accounted for in our model or false discovery rate correction. Accordingly, we leveraged the patterns of linkage disequilibrium in our inbred reference panel to infer the minimum number of unlinked loci underpinning clusters of low *p*-value SNPs throughout the genome. Specifically, we used the GLM signal obtained independently for each treatment and identified unlinked, genomic windows (hereafter, “clusters”) enriched in SNPs moving systematically throughout the experiment using a method originally described and developed by Rudman et al. (2022; source code provided: https://github.com/greensii/dros-adaptive-tracking). We then quantified the behavior of these unlinked clusters in the opposing treatment (e.g., the behavior of loci identified in HQ was then examined in LQ, and vice versa). Specifically, for each cluster, we computed the mean frequency shift of all seasonally evolving alleles (FDR < 0.1; effect size > 0.5%) in the opposing “test” treatment. To determine whether the resulting distributions of allele frequency shifts indicated parallel change across treatments, we compared them to allele frequency distributions derived from sets of matched control SNPs (i.e., each target SNP within each cluster was matched to a control SNP located on the same chromosomal arm and within 5% starting frequency in the founding population).

Finally, we used another GLM-based approach to validate evidence of SNPs with different evolutionary dynamics between treatments and throughout the experiment produced via the approaches described above. Specifically, we used coverage and population-size normalized allele frequency data across all timepoints and treatments and ran a GLM of the form: allele frequency ∼ timepoint + treatment + treatment × timepoint. We then identified those SNPs with a significant treatment by time interaction as those with an FDR-corrected *p*-value < .1. We plotted the trajectories of these treatment-by-time SNPs for each treatment throughout the experiment and visualized their genomic distribution using Manhattan plots.

## Results

### The ecological impact of variation in resource quality

Adult census estimates reveal that variation in the nutritional environment did not produce significant differences in population size across the two treatment groups, with both exhibiting similar patterns of seasonal growth throughout the summer, achievement of maximum size in early September, and subsequent autumnal decline (Figure 1). There was no overall effect of treatment on population size across the experiment (Table S2a). Furthermore, no individual pairwise comparison between treatment groups was significant at any individual timepoint after multiple testing corrections (Table S2b). These results demonstrate that, contrary to our prediction, the resource quality variation imposed here does not impact adult census size in this system.

### The impact of nutritional variation on trait evolution

**Figure 2.**
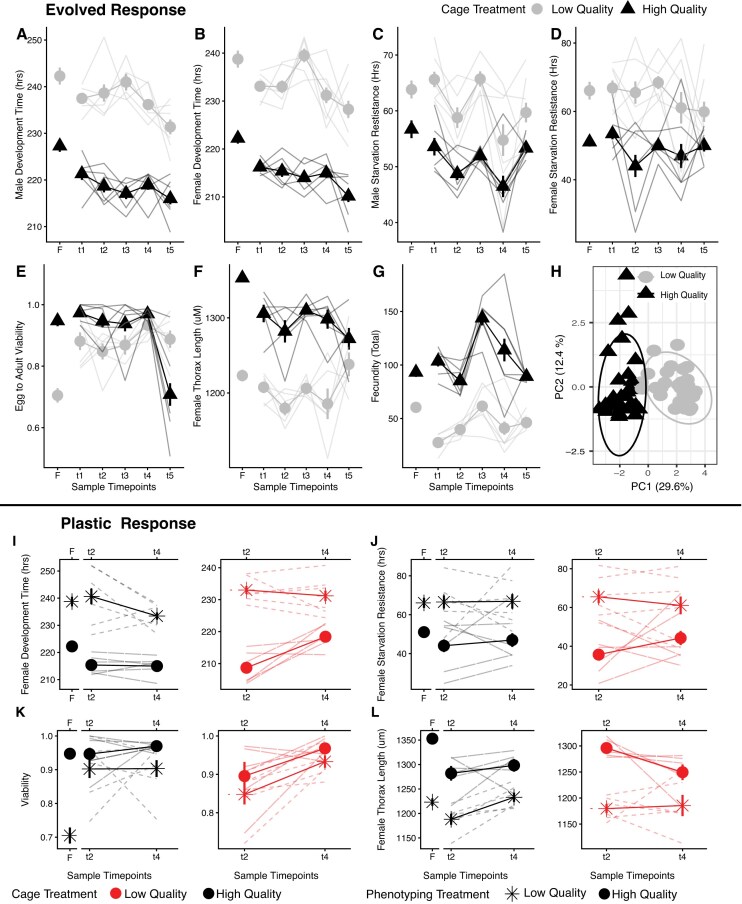
Patterns of phenotypic evolution (A–G) and phenotypic plasticity (I–L) over seasonal time in two distinct resource environments. Mean trait values (± *SE*) are depicted by bolded lines with symbols (low-quality [LQ] circles, high-quality [HQ] triangles), and individual replicate cages are given as non-bolded lines (A–G). Founder populations (sample timepoint F) are included for presentation purposes only. Variation in the nutritional environment impacts all assayed traits and shifts patterns of seasonal adaptation for two traits (E, F). When all trait means across all timepoints are used to construct a principal component analysis (H), a strong effect of nutritional environment is observed with a significant effect in a MANOVA (*F*_2,58 _= 1.28E^+02^, *p* = 2.2E^−16^). Patterns of phenotypic plasticity for HQ and LQ populations assayed on both diets at two timepoints (I–L; plastic response for all traits given in Figure S1). Mean trait values (± *SE*) are depicted by bolded lines with symbols (treatment denoted by color [LQ in red on right panels, HQ in black on left panels]), and assay treatment denoted by shape (LQ denoted by an asterisk, HQ by solid points), and individual replicate cages are given as non-bolded lines. While trait value is affected by the assay environment, there is no effect of dietary treatment on the evolution of the plastic response (Table S4).

Our longitudinal phenotypic analysis revealed patterns of rapid evolutionary change across all surveyed traits (Figure 2A–G, Figure S2, Table S3). The two dietary treatments had a pronounced and direct effect on phenotype (Figure S3), as expected: This is evident in the phenotypes exhibited by the founding population cultured on the two diets, as well as in the separation between treatments in the multivariate analysis (Figure 2G). Our primary interest, however, was to examine whether patterns of evolution over seasonal time were parallel or divergent between the two dietary treatments, as demonstrated by a significant interaction between time and treatment. We find that two traits show distinct patterns of evolutionary change between treatments over seasonal time: viability and body size (Figure S2, Table S3). Viability remained roughly consistent over time for the LQ treatment; for the HQ treatment, viability remained constant until the last sampling interval, over which it declined by approximately 30%. Similarly, body size was generally constant over time for both treatments from t1 to t4. Over the last sampling interval, however, body size evolved to be larger in the LQ treatment while continuing to decrease in the HQ treatment. These differential patterns of phenotypic evolution over seasonal time were concentrated at the last timepoint, where rates of evolution were also observed to be noticeably higher as temperatures and population sizes declined.

At two timepoints (t2 and t4), all population trait values were assayed on both diets in a common garden laboratory environment. All traits exhibited pronounced plasticity in response to the dietary environment (Figure 2I–L). However, we found no evidence that the treatment manipulation affected how nutritional plasticity changed over time (Table S4), as no three-way interaction (timepoint × mesocosm diet × phenotyping diet) was significant for any of the measured phenotypes. For two traits, however, we observed shifts in nutritional plasticity over time (viability and female development rate), suggesting that the evolution of plasticity may be an essential component of rapid adaptation to environmental change in this system.

Rates of phenotypic evolution in haldanes (H) were determined for all traits between each sampling timepoint for both treatment groups (Figure S4). Rates of phenotypic evolution increase systemically across time intervals, with the fastest rates observed in the final time interval (t4 to t5) for all phenotypes. Interestingly, when viewed across the entire experiment (t1 to t5), rates are lower; this is consistent with the observation that rates of evolution, even over the timescales examined here are determined by the measurement interval (Gingerich, 1983). We observed no strong effect of the nutritional environment on the pace of evolutionary change (Figure S4C); thus, the dietary manipulation affected evolutionary trajectories but not necessarily rates of change over generational time.

When conducting an experimental manipulation of dietary sources, we would be remiss not to address the potential effects of the dietary treatments on the microbial community (Figures S6–S9), which could, in turn, affect host phenotype and fitness. Regular sampling of the microbial community (see supplemental methods) revealed that LQ samples tended to hold more lactic acid bacteria in lab culturing and greater acetic acid bacteria in the field, relative to HQ populations that were more consistent across environments (Figure S8). Overall, the total bacterial abundance of the food substrate (Figure S7) and diversity (Figure S7)/community composition (Figure S8, Table S6) of the *D. melanogaster* host populations were not significantly affected by treatment. While this level of sequencing resolution cannot exclude the impact of subtle variations in individual taxa, these results indicate that the observed population dynamics and patterns of evolutionary change reflect a host response to the dietary treatments, rather than direct or indirect effects associated with distinct microbial communities.

### Impact of nutritional variation on genomic evolution

**Figure 3.**
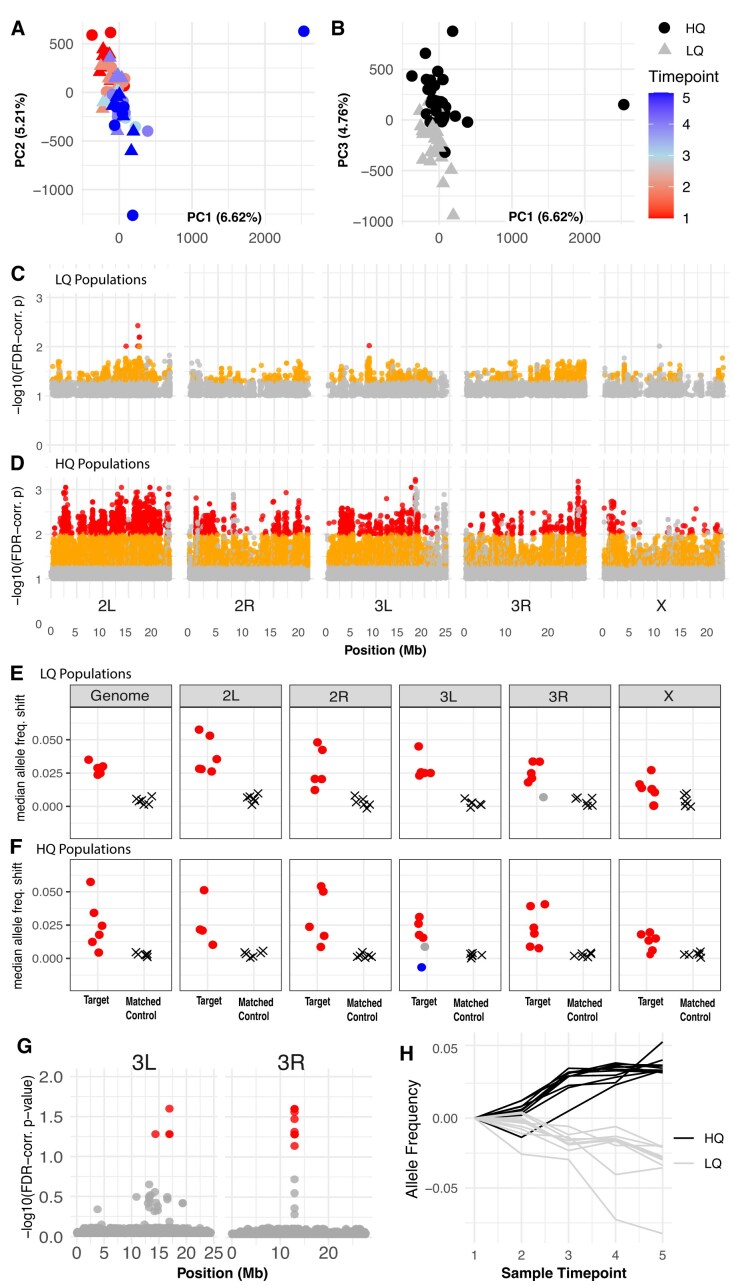
Genome-wide patterns of variation within and between treatments throughout the experiment. (A/B) Principal component analysis across all samples using allele frequency data from 1.9 million single-nucleotide polymorphisms (SNPs). In (A), samples are projected onto the first two principal components, shaped by treatment and colored by collection timepoint. MANOVA revealed a significant effect of time overall (*F* = 32.65, *p* = 5.07 × 10^−10^), as well as in the low-quality (LQ; *F* = 17.31, *p* = 2.11 × 10^−5^) and high-quality (HQ; *F* = 3.61, *p* = 3.16 × 10^−2^) populations individually, but no effect of treatment or the time by treatment interaction. Variation in PC1 is essentially driven by a single outlier sample, which is likely due to a more severe bottleneck in one cage over the t4 to t5 interval. In (B), samples are projected onto the first and third principal components and shaped by treatment. Here, the MANOVA reveals a significant effect of treatment (*F* = 25.88, *p* = 1.31E^−08^, as well as time (*F* = 5.21, *p* = 8.49 × 10^−3^). (C/D): Manhattan plots depicting SNPs shifting in parallel across replicates through time in LQ (C) and HQ (D). Point color corresponds to FDR-corrected *p*-value and effect size (gray: FDR < 0.1 and effect size > 0.5%; orange: FDR < 0.05 and effect size > 2%; red: FDR < 0.01 and effect size > 2%). (E/F): Cross-treatment validation for seasonally evolving SNPs (identified in B/C). In each treatment, seasonally evolving sites (FDR < 0.05, effect size > 2%) were identified using all six populations and were then compared to the shifts at those sites in the alternative treatment populations. The set of shifts at the rising allele was compared to shifts at background sites matched for chromosome and initial frequency. Plotted is the median shift for the matched controls (Xs), and test sites (circles), which are colored red if the test sites were significantly parallel, blue if anti-parallel, and gray if there is no significant difference from the background movement. SNPs were identified in the LQ treatment and tested in the HQ treatment populations (E), genome-wide and within each chromosome arm, and were also identified in the HQ and tested in the LQ populations (F). (G) Manhattan plot of alleles that shift differentially over time between treatments (FDR < 0.1; only chromosomal arms 3L and 3R depicted, as no significant SNPs were identified on the remaining chromosomes). Fourteen SNPs were identified with a significant treatment by time interaction via a GLM (red points). (H) Mean frequency trajectories of significant SNPs depicted in (G) throughout the experiment, separately for the HQ (black) and LQ (gray) replicate cages.

PCA indicated that the variance in allele frequencies across all samples was primarily influenced by seasonal time and secondarily by the resource environment (i.e., the sample segregation along PCs 2 and 3 corresponded with the collection timepoint and resource environment, respectively, as shown in Figure 3A, B). Our regression-based analysis further exemplified the appreciable force of seasonally varying selection. Throughout the experiment in both LQ (Figure 3C) and HQ (Figure 3D) treatments, we quantified genome-wide signatures of selection across replicates whereby thousands of SNPs (2,203 in LQ, and 29,303 in HQ), across all chromosomal arms, exhibited parallel movement (FDR < 0.05) of relatively large effect (frequency change > 2%) throughout the experiment (Figure 3C and D).

We next aimed to specifically assess the degree of parallelism in the genomic response to seasonal changes between HQ and LQ replicates (Figure 3E and F). This analysis revealed that the response to seasonal change across chromosome arms is predominantly parallel, whereby the primary direction of allele frequency movement is concordant between treatments with a magnitude of change that exceeds background allele frequency movement (red points, Figure 3E and F). Interestingly, this parallelism was reduced on 3L and 3R and, in one instance, was antiparallel (blue point, Figure 3F), providing evidence that a subset of loci may exhibit patterns of divergent seasonal responses between treatments.

We next sought to quantify the relative contribution of parallel vs. anti-parallel seasonal selection between treatments across the genome. This analysis revealed 81 clusters in HQ and 117 clusters in LQ. We then quantified the behavior of the rising allele at all GLM-significant SNPs within each cluster in the opposing treatment relative to a set of matched controls. Our findings again showed predominantly parallel shifts between treatments, with 96% of clusters in HQ and 95% in LQ exhibiting significantly parallel behavior in the opposing treatment. Notably, however, we identified one cluster in HQ and three clusters in LQ that demonstrated antiparallel movement between treatments (Figure S5, Table S7), indicating that selection was acting in opposite directions. As suggested by the analysis in Figure 3E and F, this antiparallel movement was confined to chromosome arms 3L and 3R. Overall, these results illustrate a largely parallel genomic response to seasonal changes across treatments, with distinct deviations from parallelism consistently observed. Furthermore, we found that genome-wide and chromosomal arm-specific correlation coefficients of allele frequency movement between treatments were far greater than expected by chance (all point estimates of allele frequency correlations fell outside the null distribution generated via permutations) (Figure S10A, Table S8). These correlations were particularly heightened on chromosomal arm 2L, and relatively depressed on 3L and X. We found that correlations produced using the subset of SNPs exhibiting evidence of systematic shifts among replicates independently within each treatment were significantly greater than random subsets of the empirical data, suggesting that these loci likely drive the signatures of correlated allele frequency movement between treatments (Figure S10B and C, Table S8).

Finally, we aimed to identify individual SNPs with treatment-specific trajectories. This model identified many SNPs with systematic movement across all samples through seasonal time (e.g., 16,776 SNPs at an FDR < 0.1) and 14 SNPs that displayed a significant treatment-by-time interaction (FDR < 0.1). Ten of the treatment × time SNPs are located on chromosome 3R (positions 12959498–12998860), while four are on 3L (one at position 14360958 and three others between positions 16911479–16911529; Figure 3G, Table S5). This result underscores the extent of the shared seasonal response compared to the relatively localized divergent selection between treatments over time. Visualization of the trajectories of these 14 SNPs throughout the experiment illustrates their treatment-specific dynamics. Specifically, each SNP demonstrated a magnitude of allele frequency change greater than ∼3%, with the direction of allele frequency movement being opposite between treatments in all cases. These patterns indicate a set of loci under strong selection in both nutrient environments but selected in opposing directions (Figure 3H). Collectively, the genomic data revealed pervasive seasonal evolution within each treatment, as well as evidence of localized divergent responses to selection between treatments. Therefore, we find that distinct resource environments contribute to differing patterns of evolution at both the genomic and phenotypic levels.

## Discussion

We report the observations from a large-scale, replicated field experiment in which the dietary resources of half of the populations were significantly reduced (20% fewer calories, 60% reduction in protein). Despite this difference in dietary resources, the largely parallel response within and between treatments indicates the robustness of the population response to seasonally dynamic environments. However, we also show that the trajectory of phenotypic evolution is altered by dietary treatment, as are patterns of allele frequency change at multiple regions of the genome. Given the parallelism among replicates in patterns of evolutionary change, we interpret these patterns as adaptive: diet impacts how experimental populations adapt to the changing seasonal environment.

Our dietary treatments elicited very distinct phenotypic responses in all populations, which manifested prominently when surveyed on the alternative diet. The HQ diet is a typical rich diet for laboratory culture and the diet used in prior mesocosm field experiments in this system (Bitter et al., 2024; Grainger et al., 2021; Rajpurohit et al., 2017; Rudman et al., 2019, 2022). The LQ diet was designed to mimic a natural, late-season diet in temperate environments. Natural populations of *D. melanogaster* in temperate North America persist on a wide range of substrates that vary in nutritional/caloric content and are seasonally ephemeral as well as spatially variable (e.g., strawberries, citrus, cherries, grapes, persimmons, paw paws, peaches, pears, apples, etc.). The results we present here suggest that variation in the dietary environment can alter selection pressures and produce localized variation in genomic adaptive trajectories, which may contribute to the maintenance of genetic variation in natural populations.

Ecologically, we hypothesized that resource quality would impact patterns of adult population size over time, whereby the LQ replicates would exhibit a limited rate of growth, reduced carrying capacity, and earlier extirpation relative to the HQ treatment. This was rejected, as no distinction between treatments was observed. Several potential explanations exist for these results. Our experimental populations may, in fact, not have been resource-limited by our experimentally imposed diets. However, all available food (400 ml) was consumed in 14 days, and mesocosm populations’ reproductive output during summer is ∼10,000–30,000 eggs per cage every 2 days (Rudman et al., 2022). Additionally, dietary treatment could have influenced rates of senescence and average lifespan, making population sizes similar across treatments despite different underlying age structures. Although diet affects lifespan in *D. melanogaster* (Skorupa et al., 2008; Zanco et al., 2021), estimates (Lee et al., 2008) suggest similar lifespans for both treatment groups. Treatment differences in the intensity of intraspecific competition might also explain this result: HQ populations, with higher fecundity and viability, could initially surpass LQ populations until growth is limited by competition (Connell, 1983), yet LQ census sizes exceeded HQ throughout the first 2 months. However, as reproductive output in the field was not surveyed directly, the potential effects of differences in larval density and per capita resource variation across treatments cannot be excluded. Furthermore, differences in substrate crowding due to treatment effects on body size (flies were ∼10% larger on HQ) may limit HQ population growth due to increased competition. Lastly, our dietary manipulation might have altered the microbial community, leading to indirect host effects. We found no significant differences in bacterial abundance or notable taxonomic differences between treatments, indicating that microbial-derived protein or mutualistic effects are likely equivalent across treatments. Variations in the microbial community appear insufficient to impact long-term population demographics. Regardless, our results demonstrate that variation in the resource environment does not influence the rate of population growth, maximum size, or persistence in this experimental system. We speculate that a combination of factors may contribute to this, but overall, the ecological dynamics in our system are likely primarily driven by intraspecific competition that may be modulated in multiple ways by the nutritional environment.

Evolutionarily, variation in the nutritional environment did not restrict the capacity, rate, or magnitude of the adaptive response of populations; while the response to seasonal shifts across all populations was generally robust, the dietary manipulation did cause divergence in evolutionary trajectories for a subset of phenotypes (viability and body size) and had a quantifiable impact on patterns of allele frequency shifts over the course of the experiment. The treatment divergence, both genomically and phenotypically, demonstrates that these populations can adaptively track and respond to shifts in the resource environment, as well as the seasonal environment, concurrently. We identified 14 SNPs that demonstrated opposite patterns of allele frequency change between treatments. Over half of the SNPs with treatment-by-time interactions are in a ∼40 kb window on chromosome 3R, within a region associated with inversions (3RP/3RK) that have been linked to phenotypic and adaptive divergence (Durmaz et al., 2018; Kapun et al., 2023). The remaining SNPs were found on 3L in two regions separated by 2.5 Mbp. These 14 SNPs do not represent 14 independent loci of selection: given the recombinational landscape in *D. melanogaster* (Comeron et al., 2012; Hunter et al., 2016) and the composition of the founding population, we show that this is likely driven by selection on three genomic regions with strong linkage disequilibrium in each. Overall, the divergence in the selection landscape between treatment groups suggests that the genetic architecture underlying this treatment effect is relatively simple, which stands in stark contrast to the highly polygenic seasonal adaptive response.

The highest rates of phenotypic change and the greatest divergence in trait evolution between treatment groups (Figure 2C–F) were observed between the final sampling timepoints when temperatures dipped below freezing. This suggests that selection pressures may be most pronounced during this pre-winter phase of the season, magnifying the effect of our dietary treatments. Similarly, increased rates of evolutionary change during this period (post-frost) have been previously observed (Behrman & Schmidt, 2022; Bergland et al., 2014; Bitter et al., 2024; Grainger et al., 2021), suggesting that this relatively punctuated selective landscape may be critical in establishing seasonal cycling of both phenotypic means and allele frequencies (Behrman & Schmidt, 2022; Bergland et al., 2014; Schmidt & Conde, 2006). The dynamics of overwintering and seasonal adaptation in *D. melanogaster* populations may also be strongly impacted by intense selection in the pre-winter phase, and variation in the resource environment may be especially consequential during this period (Huang et al., 2021; Kimura & Yoshida, 1995; Kimura et al., 1992; Saunders et al., 1990).

We observe a significant impact of dietary resources on the evolutionary paths of populations, with no observable effect on one metric of ecological outcome. This finding reinforces the pace of evolutionary change over ecological timescales characterized by seasonal boom and bust population dynamics. Consequently, this suggests that the interpretation of ecological metrics should consider the underlying evolutionary divergence that may not be reflected in demographic outcomes. Moreover, this implies that monitoring evolutionary changes may be an effective measure for detecting short-term population shifts, as our findings hint that it can reveal subtle alterations with greater sensitivity. *D. melanogaster* populations can adaptively track seasonal heterogeneity (Bitter et al., 2024; Rudman et al., 2022), and we now report a simultaneous adaptive response to an additional axis of environmental variation. This ability to track and respond to fine-scale variation may drive the maintenance of genetic and phenotypic diversity globally, as natural populations respond to multiple axes of variation concurrently.

## Supplementary Material

qraf031_Supplemental_File

## Data Availability

All raw data and analysis code are found at https://github.com/jkbeltz/EVL3-25-0011.R1.git and https://doi.org/10.5061/dryad.t1g1jwtfr. Raw sequencing reads associated with the allele frequency data analyzed in this experiment are publicly available as an NCBI SRA under the BioProject ID: PRJNA1306087.
