# Supplementary material for: Variation in the resource environment affects patterns of seasonal adaptation at phenotypic and genomic levels in Drosophila melanogaster
Source: Evol Lett. 2025 Sep 22;9(6):663–74. doi: 10.1093/evlett/qraf031 (PMC12676458; doi:10.1093/evlett/qraf031)
Supplement: qraf031_Supplemental_File [file qraf031_supplemental_file.docx]

# **SUPPLEMENTAL MATERIALS**

Supplemental Materials and Methods

*Founding population establishment*.

The outbred founder population was constructed from 80 fully sequenced *Drosophila melanogaster* inbred lines to facilitate the use of haplotype inference to attain high effective sequencing coverage. These inbred lines were derived from wild-caught individuals collected June 1-6, 2012, from Linvilla Orchards, Media, PA, USA (Tilk et al. 2019). Each line was subsequently inbred for more than 20 generations of full-sibling mating, during which time they were maintained at 25˚C with a 12:12 photoperiod and fed a cornmeal molasses diet (recipe found in Table S1).

To initiate the baseline population in this experiment, each line from the seasonal inbred panel was cultured (N=10 replicate vials per line) at low density (30 ± 5 eggs, by manual counting and addition/subtraction with a brush) for two generations. From each line, 10 males and 10 females from the same 24h cohort (3d of age) were collected using light anesthesia and combined into a single population cage (.3m^3^) containing 4 replicate bottles of 50ml culture medium; 4 new bottles were added every 24h for 4d and randomly assigned to 1 of 4 replicate cages. This process was repeated, doubling the amount of food each generation, for 4 generations of recombination. In the fifth generation, flies were cultured at low densities (30 ± 5 eggs) in replicate vials at 25˚C, 12L:12D. All experimental flies were collected from a single 24h cohort (2d of age) and randomly assigned to cage/treatment. Flies were transported in small cages from the laboratory to the experimental orchard and released.

*Mesocosm experimental set up*.

Each field cage is a 2m x 2m x 2m mesh enclosure around a dwarf peach tree located outdoors (Philadelphia, PA) and features a natural insect and microbial community. The ground was fresh soil with clover planted as ground cover in each cage. The only food source and egg-laying substrate was 400ml of Drosophila media contained in 900 cm^3^ aluminum loaf pans that were added every second day for the duration of the experiment (July 20th - November 15th, 2020). After two days, each loaf pan was covered with a screen mesh lid and placed in a separate small enclosure (30 cm x 30 cm x 30 cm cage) located on the bottom rack of the shelf in the corner of each enclosure. These recently laid eggs were allowed to develop until eclosion ceased, and adults were released from each small cage every time new food was introduced to the enclosures. Loaf pans of media within experimental cages were protected from rain and direct sunlight on the second shelf of each cabinet, which was oriented away from direct sunlight.

*Phenotypic assays.*

To assess phenotypic evolution over the course of the experiment, at each sampling timepoint we collected ~2500 eggs overnight from each cage on a fresh supply of food (400ml respective diet), brought them to the laboratory, and reared them for an additional generation in a common garden (25˚C, 12L:12D) while maintaining population sizes at ~2500 individuals (Behrman *et al.* 2015; Behrman & Schmidt 2022; Rudman *et al.* 2022). Fitness-associated phenotypes were measured on 3-5 day old adults maintained at densities of 30 +\- 5 individuals manually counted and scraped from food surface into narrow vials (Fly Stuff 32-116), in the F2 generation, with 10 replicate vials per replicate field cage. Eggs used to found each of these phenotyping replicates were collected by placing fresh loaf pans into each population's F1 generation (eggs collected from field) individuals were aged 2 days after eclosion. Replicates were housed in their experimental units (9 ml of respective diet per vial) before assaying and were mated prior to sexing, then separated by sex and placed in the assay environment. Individuals were sorted using CO_2_ anesthesia and were given twenty-four hours to recover prior to assaying. Fecundity was measured as the total number of eggs laid by a group of five females, counted each day for a period of three days, with four replicate vials for each cage at each time (Paaby *et al.* 2014). Larval development rate was tracked as the time from when eggs were laid until eclosion in three replicate vials from each cage at each time point, with 50 eggs in each vial (Behrman *et al.* 2015). Viability was measured as the total number of successful adults that emerged from each of the three replicates, each consisting of 50 eggs.  Starvation tolerance was measured as the time to starvation in three replicate vials containing a 1 % agar solution for 10 males or 10 female flies across three replicates for each cage at each time point. Thorax length (a proxy for body size), was measured as the longest length across the dorsal shield in lateral view for 15 ethanol-preserved females from each population. Measurements were recorded using a Leica MZ9.5 microscope, with an Olympus DP73 camera and CellSens standard measuring software (Betancourt et al., 2021). All replicates across all generations and during phenotyping assays were maintained under common garden laboratory conditions (25˚C, 12L:12D).

Reciprocal phenotyping of all populations on both diets, under the same standardized conditions of 25˚C, 12L:12D, was conducted at t2 and t4. The phenotyping protocol was implemented in the same manner as all other common garden phenotyping. After F1s collected from the field populations were reared in the lab, eclosed and aged for 2 days, 30 +/- 5 eggs were manually scraped from the food surface and placed into ten replicate vials for both the LQ and HQ diet. Vials containing eggs and their respective diets were maintained in laboratory conditions and phenotyped in the same manner as described above.

*Phenotypic Statistical analysis*

To test for parallel phenotypic evolution in each of the six phenotypes we carried out separate linear mixed effect models and tested for significance using ANOVA (lme4 and R, respectively). Q-Q plots and the Shapiro test were used to confirm that the data for a given phenotype fit a normal distribution. Starvation resistance rate, development rate, and body size fit a normal distribution without transformation, whereas viability (as a percentage) required an arcsine square root transformation, and fecundity a log transformation, in order to effectively fit a normal distribution. Lmer () function in the lme4 package was used to model the data (Bates *et al.* 2015).

For common garden phenotyping at all five timepoint throughout the experiment the model accounted for the effect of time, treatment and the interaction term while treating individual cage replicate as a random effect:

Phenotype ~ Treatment + Timepioint + Treatment:Timepoint + (1| Replicate #)

When reciprocal common garden culture was conducted on both diets at t2 and t4, this model was expanded to account for the predictors and interactions on assay diet:

Phenotype ~ Treatment + Timepioint + Assay Diet + Treatment: Assay Diet + Treatment:Timepoint + Assay Diet : Timepoint + Assay Diet:Timepoint:Treatment + (1| Replicate #)

Finally, when the effect of treatment on census size was estimated with a mixed model GLM, again histograms and Q-Q plots were used to confirm that a normal distribution fits the census data, and the model was constructed as follows:

Census size ~ Treatment + Timepioint + Treatment:Timepoint + (1| Replicate #).

## *Microbial Sampling and Sequencing Methods*

Since the Drosophila-associated microbiota shifts over space and time (Chandler *et al.* 2011; Wong *et al.* 2013), affects host phenotypes (Chandler *et al.* 2011; Keebaugh *et al.* 2018; Wong *et al.* 2014), and varies with dietary substrate (Massey *et al.* 2024; Yakovleva *et al.* 2024), we examined the host and environmental microbiota in all experimental populations at three timepoints. On September 18, October 25 and November 14, approximately 100 adults were directly sampled by aspiration from each cage for microbial sequencing. Additionally, a sample of older food (which has been in the environment for at least 10 days, and still had flies actively eclosing) was collected from each cage to assess the microbial community associated with each dietary substrate. These samples, along with common-garden F1s from phenotyping samples, were stored at -80˚C; upon completion of the experiment, DNA was extracted from three replicate pools of five male and five female flies, and amplicon libraries were generated using 16s rRNA V1V2 primers (Forward: 5'-AGAGTTTGATCCTGGCTCAG-3', Reverse: 5'-TGCTGCCTCCCGTAGGAGT-3') and Q5 polymerase before being paired-end sequenced with Illumina MiSeq to 15x coverage at the University of Pennsylvania / Children’s Hospital of Philadelphia microbiome core, following previously described protocols (López-Aladid *et al.* 2023). All reads identified as Wolbachia sp. were excluded from the analysis. All sequence analysis was performed using QIIME2 (following code provided at<https://github.com/jbisanz/16Spipelines>) and visualizations generated with R (version 4.3.2). Raw sequence data are available at <https://github.com/jkbeltz/EVL3-25-0011.R1.git> & <https://doi.org/10.5061/dryad.t1g1jwtfr>.

# **SUPPLEMENTAL FIGURES AND TABLES**

### **Table S1:**

Nutritional composition for both dietary treatments. The lab standard diet (HQ) consists of 81.0% distilled water, 8.0% molasses (Golden Barrel Sulfur-Free Black Strap Molasses), 5.3% cornmeal (Flystuff 62-100), 3.0% ethanol, 1.5% inactive dry yeast (Flystuff 62-108), 0.73% soy flour (Flystuff 62-115), 0.5% agar (Flystuff 66-103), and 0.17% methylparaben (Apex Bioresearch Products 20-258) by weight. The semi-natural apple-based diet (LQ) consists of 55.3% unsweetened applesauce (Regal #10 Can Unsweetened Applesauce), 39.0% distilled water, 3.0% ethanol, 1.5% inactive dry yeast (Flystuff 62-108), 0.65% agar (Flystuff 66-103), and 0.17% methylparaben (Apex Bioresearch Products 20-258) by weight. Preparation of both the LQ and HQ diets followed the same cooking protocol. Macronutrient calculations were done using the Drosophila Dietary Composition Calculator (<https://brodericklab.com/DDCC.php>).

| **Nutritional Facts / 100 ml** | **Cornmeal Molasses Diet (High Quality)** | **Apple Diet (Low Quality)** |
| --- | --- | --- |
| Calories | 64.52 | 47.18 |
| Fiber (g) | 1.43 | 2.409 |
| Sugars (g) | 5.91 | 9.016 |
| Protein (g) | 1.73 | 0.43 |
| Fat (g) | 0.45 | 0.1 |
| Carbohydrates (g) | 14.99 | 7.95 |
| Protein: Carbohydrates | 1:8 | 1:14 |

**Figure S1:** Experimental diagram depicting the experiment mesocosms and sampling regime over time. Six replicate mesocosms were used for each dietary treatment, and sampled at five time points (8/12, 9/18, 10/3, 10/25, 11/15,). Common garden phenotyping on the diet of origin and poolseq genotyping of all replicate populations was conducted at five timepoints. At two timepoints (t2 and t4), populations were assayed on both diets in a reciprocal common garden design (see methods and supplemental methods). Microbial surveys of the host (field-collected and F1) and environment (food substrate) were conducted at three time points (t2, t4 & t5).

### **Table S2a & b**

A linear mixed-effects model was constructed to determine the effect of nutritional quality on census size across replicate mesocosms. The model was tested across all 11 timepoints, and pairwise comparisons were made across the treatment group at each timepoint. Normality of the data was confirmed using Q-Q plots and Shapiro-wilks test (p=.102). ANOVA was used to assess the impact of sampling time and treatment on census size across all phases of the experiment. The model was constructed in R using lme4 (Bates *et al.* 2015), and ANOVA was used for post hoc analysis (see supplemental methods).

Linear mixed model fit by REML. t-tests use Satterthwaite's method ['lmerModLmerTest'],

1. 𝑦𝑖𝑗=𝛽0+𝛽1𝑋1𝑖𝑗+𝛽2𝑋2𝑖𝑗 +𝛽4𝑋1𝑖𝑗𝑋2𝑖𝑗+𝑢𝑖+𝜀𝑖𝑗

Response Variable (𝑦 = Pop Size)

Fixed effects (𝑋1 = Timepoint (1:11) , 𝑋2 = Cage Treatment (LQ vs. HQ) )

Random Effects (𝑢 = Cage Replicate,)

| Comparison | Sum Sq. | Mean Sq | DF | F-value | p-value |
| --- | --- | --- | --- | --- | --- |
| TIme | 0.721 | 0.721 | 11,110 | 38.35 | <.0001 |
| Treatment | 0.543 | 0.543 | 1,10 | 0.126 | 0.7293 |
| Time:Treatment | 0.236 | 0.236 | 11,110 | 0.628 | 0.8011 |

1. Pairwise comparison of treatment groups at each timepoint from the model above. No significant effect treatment at any individual timepoint across the seasonal experiment. DF=10

|  |  | |
| --- | --- | --- |
| Timepoint | t-ratio adjusted p value | |
| 7/21/20 | -4.23 x 10^-15^ | 1.00 |
| 8/14/20 | 0.836 | 0.423 |
| 8/31/20 | -0.264 | 0.797 |
| 9/4/20 | 1.34 | 0.211 |
| 9/11/20 | 0.895 | 0.392 |
| 9/18/20 | 9.97 x 10^-2^ | 0.923 |
| 9/25/20 | 1.24 | 0.242 |
| 10/9/20 | -0.755 | 0.467 |
| 10/18/20 | -0.492 | -0.633 |
| 11/1/20 | -0.242 | 0.814 |
| 11/10/20 | -0.444 | 0.666 |
| 11/21/20 | -3.40 x 10^-2^ | 0.974 |

### **Table S3:**

A linear mixed-effects model was constructed to determine if treatment (LQ & HQ), season (t1-5), or the interaction of both has effects on life history traits. Trait value is the response; treatment, timepoint, and the interaction term are predictors (individual cage treated as a random effect). Models were constructed for each trait individually in R using lme4, and anova() was used for post hoc analysis; additional details are found in supplemental methods.

Linear mixed model fit by REML. t-tests use Satterthwaite's method ['lmerModLmerTest']

𝑦𝑖𝑗=𝛽0+𝛽1𝑋1𝑖𝑗+𝛽2𝑋2𝑖𝑗 +𝛽4𝑋1𝑖𝑗𝑋2𝑖𝑗+𝑢𝑖+𝜀𝑖𝑗

Response Variable (𝑦 = Phenotype )

Fixed effects (𝑋1 = Timepoint (1:5) , 𝑋2 = Cage Treatment (LQ vs. HQ) )

Random Effects (𝑢 = Cage Replicate,)

|  | Comparison | Sum Sq. | Mean Sq | DF (num, den) | F-value | p-value |
| --- | --- | --- | --- | --- | --- | --- |
| Male Larval Development Time | Time | 118.92 | 118.92 | 1,56 | 9.55 | 3.1 x 10^-4^ |
|  | Treatment | 78.2 | 78.2 | 1,56 | 54.12 | <1.0 x 10^-4^ |
|  | Time: Treatment | 1.12 | 1.12 | 1,56 | 0.279 | 0.5989 |
| Female Larval Development Time | Time | 65.3 | 65.3 | 1,56 | 7.64 | 7.6 x 10^-3^ |
|  | Treatment | 83.2 | 83.2 | 1,56 | 41.71 | <1.0 x 10^-4^ |
|  | Time: Treatment | 36.1 | 36.1 | 1,56 | 1.6 x 10^-2^ | 0.899 |
| Male Starvation Resistance | Time | 74.8 | 74.8 | 1,56 | 2.78 | 0.101 |
|  | Treatment | 53.15 | 53.15 | 1,57 | 14.28 | <1.0 x 10^-4^ |
|  | Time: Treatment | 15.34 | 15.34 | 1,58 | 1.36 | 0.2476 |
| Female Starvation Resistance | Time | 72.26 | 72.26 | 1,56 | 1.42 | 0.2385 |
|  | Treatment | 105.2 | 105.2 | 1,56 | 10.08 | 2.4 x 10^-3^ |
|  | Time: Treatment | 61.61 | 61.61 | 1,56 | 0.582 | 0.4487 |
| Thorax Length (Body Size) | Time | 36.1 | 36.1 | 1,56 | 3.5 x 10^-2^ | 0.8636 |
|  | Treatment | 112.5 | 112.5 | 1,56 | 41.29 | <1.0 x 10^-4^ |
|  | Time: Treatment | 58.7 | 58.7 | 1,56 | 3.99 | 0.0501 |
| Fecundity (Log transformed) | Time | 11.2 | 11.2 | 1,56 | 0.691 | 0.4094 |
|  | Treatment | 87.5 | 87.5 | 1,56 | 23.70 | <1.0 x 10^-4^ |
|  | Time: Treatment | 44.7 | 44.7 | 1,56 | 0.677 | 0.414 |
| Percent Viability (arcsine transformed) | Time | 0.249 | 0.249 | 1,56 | 5.984 | 0.017 |
|  | Treatment | 0.723 | 0.723 | 1,56 | 17.33 | <1.0 x 10^-4^ |
|  | Time: Treatment | 0.514 | 0.514 | 1,56 | 12.32 | <1.0 x 10^-4^ |

**Figure S2*.*** Mean (± one s.e.) phenotypes for replicate populations (N=6) reared on the low-quality (LQ, red) and high-quality (HQ, black) nutritional environments and assayed on LQ (asterisk/dash) and HQ (solid) environments at two experimental time points (mid-September (t2) & early November (t4)). Thinner dashed and solid lines represent individual replicate populations. Assaying each population in both treatment environments allowed us to determine if the plastic response to diet is evolving differentially across treatments and/or over seasonal time. The founder population, which was reared on the HQ diet, was also assayed in both environments, and is included purely for presentation purposes. We find that across treatments and time, assay diet primarily determines trait value. The mixed-effect linear model (Table S4) found that the phenotyping treatment (assay diet) significantly impacted all mean trait values, and some traits also showed an interaction between assay diet and time. We find no indication that the plastic response is evolving differentially across treatment groups and over time.

**Table S4 :**

A three-way linear mixed-effects model was constructed to determine whether treatment (LQ vs. HQ), seasonal time (t2 vs. t4), assay diet (LQ vs. HQ), as well as any interaction terms, explain the patterns of variation across all life history traits. The model treated individual cage values and sampling timepoint as random effects. This model was constructed for each trait variable, and an ANOVA post hoc was conducted on each trained model. F-values and P-values are reported, and significant P-values are bolded. The model was constructed in R using lme4, with post hoc testing using anova(). As described in Fig S1, trait values were strongly driven by assay diet and the interaction of time and assay diet. Very little consistent effect of treatment was detected, and there is very little explanation offered by the interaction terms, showing that the plastic response is not evolving differentially across treatment and timepoint; additional details are found in supplemental methods.

Linear mixed model fit by REML. t-tests use Satterthwaite's method ['lmerModLmerTest']

𝑦𝑖𝑗=𝛽0+𝛽1𝑋1𝑖𝑗+𝛽2𝑋2𝑖𝑗 +𝛽4𝑋1𝑖𝑗𝑋2𝑖𝑗+𝑢𝑖+𝑣𝑗+𝜀𝑖𝑗

Response Variable (𝑦 = Phenotype )

Fixed effects (𝑋1 = Timepoint, X2= Assay Diet, 𝑋3 = Cage Treatment)

Random Effects (𝑢 = Cage Replicate, )

|  | Comparison | Sum Sq. | Mean Sq | DF (num, den) | F-value | p-value |
| --- | --- | --- | --- | --- | --- | --- |
| Male Larval Development TIme | Time | 115.2 | 115.2 | 1,10 | 0.031 | 0.863 |
|  | Assay Diet | 87.6 | 87.6 | 1,20 | 45.7 | <1.0 x 10^-4^ |
|  | Treatment | 178.5 | 178.5 | 1,10 | 0.76 | 0.403 |
|  | Time: Assay Diet | 92.6 | 92.6 | 1,20 | 4.14 | 0.055 |
|  | Time:Treatment | 81.7 | 81.7 | 1,10 | 6.56 | 0.028 |
|  | Treatment : Assay Diet | 58.2 | 58.2 | 1,20 | 1.3 x 10^-2^ | 0.909 |
|  | Time: Treatment: Assay Diet | 103.1 | 103.1 | 1,20 | 3.28 | 0.0612 |
| Female Larval Development TIme | Time | 113.2 | 113.2 | 1,10 | 0.12 | 0.9247 |
|  | Assay Diet | 95.6 | 95.6 | 1,20 | 1.80 | 0.131 |
|  | Treatment | 146.8 | 146.8 | 1,10 | 2.71 | 0.131 |
|  | Time: Assay Diet | 6.5 | 6.5 | 1,20 | 9.28 | 0.0064 |
|  | Time:Treatment | 71.9 | 71.9 | 1,10 | 6.64 | 0.0276 |
|  | Treatment : Assay Diet | 51.9 | 51.9 | 1,20 | 1.21 | 0.287 |
|  | Time: Treatment: Assay Diet | 77.9 | 77.9 | 1,20 | 0.61 | 0.445 |
| Male Starvation Resistance | Time | 24.6 | 24.6 | 1,10 | 3.1 x 10^-2^ | 0.8635 |
|  | Assay Diet | 32.1 | 32.1 | 1,20 | 45.77 | <1.0 x 10^-4^ |
|  | Treatment | 44.1 | 44.1 | 1,10 | 0.7624 | 0.403 |
|  | Time: Assay Diet | 6.3 | 6.3 | 1,20 | 4.144 | 0.0552 |
|  | Time:Treatment | 41.2 | 41.2 | 1,10 | 6.56 | 0.0283 |
|  | Treatment : Assay Diet | 13.7 | 13.7 | 1,20 | 1.3 x 10^-2^ | 0.909 |
|  | Time: Treatment: Assay Diet | 112.1 | 112.1 | 1,20 | 3.28 | 0.061 |
| Female Starvation Resistance | Time | 31.7 | 31.7 | 1,10 | 0.269 | 0.6075 |
|  | Assay Diet | 19.5 | 19.5 | 1,20 | 39.59 | <1.0 x 10^-4^ |
|  | Treatment | 17.7 | 17.7 | 1,10 | 0.891 | 0.367 |
|  | Time: Assay Diet | 20.1 | 20.1 | 1,20 | 1.215 | 0.279 |
|  | Time:Treatment | 31.7 | 31.7 | 1,10 | 4.0 x 10^-3^ | 0.950 |
|  | Treatment : Assay Diet | 23.7 | 23.7 | 1,20 | 9.2 x 10^-2^ | 0.764 |
|  | TIme: Treatment: Assay Diet | 11.6 | 11.6 | 1,20 | 0.5419 | 0.467 |
| Thorax Length (Body Size) | Time | 178.5 | 178.5 | 1,10 | 0.26 | 0.618 |
|  | Assay Diet | 98.6 | 98.6 | 1,20 | 104.1 | <1.0 x 10^-4^ |
|  | Treatment | 213.7 | 213.7 | 1,10 | 5.11 | 0.0473 |
|  | Time: Assay Diet | 194.7 | 194.7 | 1,20 | 5.95 | 0.0242 |
|  | Time:Treatment | 201.5 | 201.5 | 1,10 | 6.42 | 0.0296 |
|  | Treatment : Assay Diet | 178.8 | 178.8 | 1,20 | 0.45 | 0.536 |
|  | Time: Treatment: Assay Diet | 191.5 | 191.5 | 1,20 | 0.54 | 0.485 |
| Fecundity (Log transformed) | Time | 12.8 | 12.8 | 1,10 | 6.47 | 0.015 |
|  | Assay Diet | 11.9 | 11.9 | 1,20 | 62.9 | <1.0 x 10^-4^ |
|  | Treatment | 20.6 | 20.6 | 1,10 | 0.098 | 0.760 |
|  | Time: Assay Diet | 12.8 | 12.8 | 1,20 | 3.86 | 0.0585 |
|  | Time:Treatment | 21.5 | 21.5 | 1,10 | 2.1 x 10^-3^ | 0.960 |
|  | Treatment : Assay Diet | 303.6 | 303.6 | 1,20 | 0.705 | 0.408 |
|  | Time: Treatment: Assay Diet | 37.1 | 37.1 | 1,20 | 0.21 | 0.643 |
| Percent Viability (arcsine transformed) | Time | 0.972 | 0.972 | 1,10 | 0.21 | 0.718 |
|  | Assay Diet | 1.56 | 1.56 | 1,20 | 113.1 | <1.0 x 10^-4^ |
|  | Treatment | 0.894 | 0.894 | 1,10 | 1.09 | 0.319 |
|  | Time: Assay Diet | 0.792 | 0.792 | 1,20 | 1.4 x 10^-2^ | 0.905 |
|  | Time:Treatment | 0.821 | 0.821 | 1,10 | 3.226 | 0.0825 |
|  | Treatment : Assay Diet | 1.22 | 1.22 | 1,20 | 0.151 | 0.699 |
|  | Time: Treatment: Assay Diet | 0.926 | 0.926 | 1,20 | 0.229 | 0.635 |


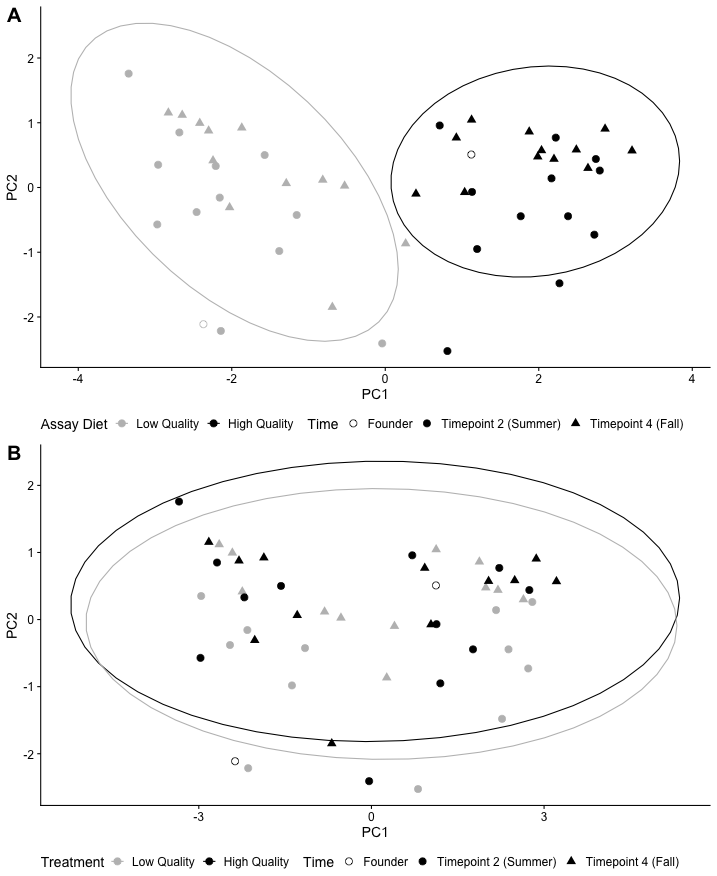


**Figure S3.** Principal component analysis was constructed using mean trait values across seven assayed traits (Fig. S2) of replicate populations (N=6) of *D. melanogaster*, reared in two distinct resource environments, collected at two timepoints, and assayed in both dietary environments. When points are shaded by the assay diet (A), we see a clear separation and a significant effect of the assay diet (F_2,47_=123, p=2.2x10^-16^). When the same points are shaded not by assay diet but by mesocosm treatment (B), we see complete overlap in the groupings (F_2,47_=0.636, p=0.534). This further demonstrates that when populations are assayed in both respective environments, the assay environment drives the trait values and that the plastic response to resource variation is not evolving differentially across treatments.

**Figure S4.** Mean rates of phenotypic evolution in haldanes were calculated for all traits between each sampling interval (A & B). Panel A shows rates of evolution for each combination of trait and time interval averaged across all replicate populations for each dietary treatment, without standard error bars for visual purposes only. Panel B shows the same data, log-transformed to better spread the data points, and includes standard error bars around the mean values. When traits are combined (C). The highest rates of trait change are consistently observed in the final time interval, where rates approach a standard deviation change per generation. The number of generations per time interval was estimated using a degree day model (see methods) and was determined to be 3.15 (Founder – t1), 2.28 (t1-t2), 1.26 (t2-t3), 1.3 (t3-t4), and 0.10 (t4-t5).

### ****

### **Fig S5:** Quantification of cross-treatment parallelism using clusters of unlinked loci enriched in SNPs identified in Fig. 3. (A) corresponds to clusters identified in HQ and tested within LQ, while (B) corresponds to clusters identified in LQ and tested in HQ. Each bar corresponds to an unliked cluster, which is shaded by the magnitude of allele frequency change quantified in the opposing treatment. Colors describe the level of parallelism between clusters on a spectrum from red to white to blue, with the darkest red (shift > 0.03) indicating significant parallelism and blue (with an asterisk) a significant antiparallel relationship between the allele frequency response. White shades indicate neutrality (no change compared to background movement), and median shades of red and blue describe the relative parallelism / anti-parallelism at these clusters, which have borderline or insignificant relationships.

### **Table S5 :**

SNPs and genes associated with divergent selection between the HQ and LQ treatment groups. These SNPs showed a significant interaction between time and treatment in the GLM. Locations are listed in *D. melanogaster* genome version r5 dm3.

| **SNP position** | **Gene Identity** |
| --- | --- |
| 3R:12959498 | None |
| 3R:12960332 | CG31267 |
| 3R:12960369 | CG31267 |
| 3R:12961464 | None |
| 3R:12961492 | None |
| 3R:12961501 | None |
| 3R:12962391 | CG5246 |
| 3R:12963490 | None |
| 3R:12964265 | CG5255 |
| 3R:12998860 | Mur89F |
| 3L:14360958 | fz |
| 3L:16911479 | Lmpt |
| 3L:16911491 | Lmpt |
| 3L:16911529 | Lmpt |

**Figure S6.** Alpha diversity metrics for bacterial communities in all populations and timepoints. Bacterial richness and diversity (Shannon & Faith Phylogenetic Distance) for field-collected adults, food supplies, and lab-reared F1s at t2 (summer-evolved) and t4 (fall-evolved). Seasonality drives all alpha diversity metrics in field samples and F1-reared adult flies, with no significant separation in treatment means at either timepoint. For the cage food supply, we see greater richness and diversity in the HQ treatment in t2 and a convergence in t4, relative to diversity/richness in the LQ treatment. The nutritional manipulation had no effect on the microbial diversity of the host in either the field or the lab. This suggests that observed patterns of phenotypic and genomic evolutionary change are likely driven directly by nutritional manipulation, rather than an indirect effect of microbial diversity. Furthermore, the fact that the manipulation altered the food supply with no effect on bacterial associations suggests that the host largely controls the composition of its microbiome (top-down) as opposed to being dictated by the environment (bottom-up).

 **Figure S7.** Principal component analysis was constructed using the relative abundance of all nonsingleton bacterial taxa in unweighted (a) and weighted (b) unifrac distance. Sequence variants were identified in field collected flies, cage food, and F1 flies reared in a single generation in a lab common garden, at experimental t2 (Summer Evolved, solid dots) and t4 (Fall Evolved, asterisks) for both LQ (grey) and HQ (black) populations. The MANOVA for significance in centroid position found no effect of nutritional quality on bacterial community composition and no effect of treatment, or the interaction term, for either unifrac distances in all samples.

### **Table S6 :**

To assess whether the variance in the bacterial communities is significant between treatment groups for all sample types, we conducted MANOVA tests on the first two axes of variation for the weighted and unweighted Unifrac distance matrices constructed using the relative abundance of all non-singleton sequence variance. Like the alpha diversity metrics, the only clear effect on the microbial communities was observed in the cage food supply samples, which were significantly shaped by the treatment group. The bacterial communities of field-collected and F1 common garden flies showed no effect from cage treatment, time, or the interaction term. These findings again suggest that the ecological and evolutionary effects observed due to the treatment are not driven by variation in the microbial communities between treatments.

Response Variable - Weighted and Unweighted Unifrac Matrices, PC1 & PC2

Fixed effects - Sample Timepoint (t2 Vs. t4), Cage Treatment (LQ Vs. HQ)

| Sample Type | Parameters  (DF num, den) | Time (2,19) | | Cage Treatment (2,19) | | Time : Cage Treatment (2,19) | |
| --- | --- | --- | --- | --- | --- | --- | --- |
|  | Test | F-Value (p-value ) | | F-Value (p-value ) | | F-Value (p-value ) | |
| Cage Food Supply | Unweighted Unifrac Distance | 1.91 | 0.176 | 2.05 | 0.157 | 2.34 | 0.124 |
|  | Weighted Unifrac Distance | 1.30 | 0.295 | 4.29 | 0.029 | 2.63 | 0.099 |
| F1 Common Garden Flies | Unweighted Unifrac Distance | 3.34 | 0.058 | 0.391 | 0.682 | 0.065 | 0.938 |
|  | Weighted Unifrac Distance | 2.96 | 0.364 | 0.193 | 0.826 | 3.07 | 0.070 |
| Field Collected Flies | Unweighted Unifrac Distance | 1.23 | 0.314 | 2.97 | 0.068 | 0.163 | 0.851 |
|  | Weighted Unifrac Distance | 1.12 | 0.348 | 2.15 | 0.101 | 0.813 | 0.458 |

 **Figure S8.** Total abundance of bacteria per sample across treatment, season, and sample type. Sequence variants were identified in wild-collected flies, cage food, and F1 flies reared for a single generation in a lab common garden, at experimental t2 (Summer Evolved, solid dots) and t4 (Fall Evolved, asterisks), for both Low Quality (LQ, Grey) and High Quality (HQ, Black) populations. The sequencing procedure and analysis protocol can be found in the methods. Raw counts of bacterial reads per sample were adjusted for the DNA concentration determined immediately after extraction to account for differences in DNA volume per sample. Seasonal change from summer to fall causes a decline in bacterial abundance in the food supply and an increase within wild-collected adults. T-tests revealed no significant effect of treatment on total bacterial abundance for wild adults and food at either time point. However, the HQ F1 common garden flies consistently have a greater abundance of bacteria than the LQ populations (p=4.21E-02). This result suggests that field treatment may impact how fly hosts take up bacterial taxa when brought back to a lab setting. Critically, the lack of effect of treatment on total bacterial abundance reveals that the volume of bacterial cells present on the food substrate is consistent across the two dietary treatments.

**Figure S9.** This heatmap illustrates the relative abundance of the ten most abundant bacterial taxa identified within and around the 12 experimental populations of D. melanogaster raised in one of two distinct nutritional environments. Major microbial groups are represented as rows, with each replicate cage as a column. HQ treatment samples are displayed on the left, while LQ samples are on the right, organized by timepoint (t2 and t4). Bacterial sequence variants were identified from samples of field-collected flies (top panels), cage food (middle), and flies raised for a single generation in a common garden laboratory environment (bottom). Bacterial sequence variants are classified by Phylum, Class, and then the lowest taxonomic groups identified with that variant (primarily Genus and species). The sequencing process and analysis protocol can be found in the methods.

We observe a generally high level of consistency in the most dominant microbial taxa across treatment groups and time, with two notable exceptions. First, Lactobacillus are virtually absent in the field-collected flies from both treatments and timepoints, as well as in the HQ treatment samples from the food supply and F1 laboratory flies. In contrast, Lactobacillus are more abundant in the LQ treatment samples from the cage food and F1 laboratory flies at both timepoints. Second, field-collected flies from the LQ treatment show a higher prevalence of Acetobacter taxa than those collected from the HQ treatment, a pattern that is consistent across the two sampling timepoints. Interestingly, Acetobacter is found at low abundance and prevalence in the F1 and cage food samples at both timepoints. We also observe a greater prevalence of Gluconobacter taxa in the LQ populations, especially in the later sampling timepoint. As expected, we see clear differences between field and lab host samples (Ludington & Ja 2020). When LQ and HQ samples are brought into a standardized lab environment, flies from 10 out of 12 LQ cages acquire Lactobacillus, while only 1 of 12 from HQ do the same. This suggests that the fly populations are evolving differently between dietary treatments and are “filtering” microbes in distinct ways. Overall, the total bacterial communities are not significantly different between treatments when flies are sampled directly from the field. However, given the level of sequencing resolution employed here, it is impossible to exclude the potential effects of treatment and seasonal evolution on individual bacterial taxa and strains.

**Table S7**: Two-tailed paired T-test results comparing allele frequency shifts for sets of SNPs identified via GLM independently in each treatment groups (see methods) in the respective treatment, relative to a set of matched control SNPs (Fig 3 E&F). These tests were used to determine whether allele frequency change between timepoints 1 and 5 in each treatment exceeded background shifts at control sites and whether the dominant direction of movement was conserved in the test treatment (FDR < 0.05).

| **Identification treatment** | **Test treatment** | **N**  **SNPs** | **Test**  **Cage** | **Chrom.** | **Median**  **Target SNPs** | **Median**  **Matched**  **SNPs** | **FDR** |
| --- | --- | --- | --- | --- | --- | --- | --- |
| **LQ** | HQ | 2203 | 1 | Genome | 0.025 | 0.004 | 1.45E-88 |
| **LQ** | HQ | 2203 | 2 | Genome | 0.029 | 0.002 | 1.95E-152 |
| **LQ** | HQ | 2203 | 3 | Genome | 0.035 | 0.006 | 7.32E-180 |
| **LQ** | HQ | 2203 | 4 | Genome | 0.030 | 0.008 | 4.50E-71 |
| **LQ** | HQ | 2203 | 5 | Genome | 0.024 | 0.001 | 4.10E-122 |
| **LQ** | HQ | 2203 | 6 | Genome | 0.029 | 0.005 | 1.19E-111 |
| **HQ** | LQ | 29303 | 1 | Genome | 0.012 | 0.001 | 0.00E+00 |
| **HQ** | LQ | 29303 | 2 | Genome | 0.004 | 0.002 | 2.68E-55 |
| **HQ** | LQ | 29303 | 3 | Genome | 0.034 | 0.005 | 0.00E+00 |
| **HQ** | LQ | 29303 | 4 | Genome | 0.057 | 0.003 | 0.00E+00 |
| **HQ** | LQ | 29303 | 5 | Genome | 0.024 | 0.003 | 0.00E+00 |
| **HQ** | LQ | 29303 | 6 | Genome | 0.018 | 0.003 | 0.00E+00 |
| **LQ** | HQ | 938 | 1 | 2L | 0.028 | 0.004 | 3.72E-52 |
| **LQ** | HQ | 278 | 1 | 2R | 0.012 | 0.001 | 4.13E-08 |
| **LQ** | HQ | 372 | 1 | 3L | 0.045 | 0.003 | 4.18E-50 |
| **LQ** | HQ | 504 | 1 | 3R | 0.018 | 0.007 | 9.65E-04 |
| **LQ** | HQ | 111 | 1 | X | 0.017 | 0.002 | 1.31E-15 |
| **LQ** | HQ | 938 | 2 | 2L | 0.035 | 0.006 | 1.47E-75 |
| **LQ** | HQ | 278 | 2 | 2R | 0.021 | -0.002 | 7.71E-16 |
| **LQ** | HQ | 372 | 2 | 3L | 0.025 | -0.001 | 1.12E-26 |
| **LQ** | HQ | 504 | 2 | 3R | 0.034 | 0.000 | 7.67E-53 |
| **LQ** | HQ | 111 | 2 | X | 0.001 | 0.000 | 5.91E-01 |
| **LQ** | HQ | 938 | 3 | 2L | 0.053 | 0.007 | 2.49E-114 |
| **LQ** | HQ | 278 | 3 | 2R | 0.042 | 0.005 | 6.84E-34 |
| **LQ** | HQ | 372 | 3 | 3L | 0.026 | 0.003 | 2.04E-25 |
| **LQ** | HQ | 504 | 3 | 3R | 0.021 | 0.006 | 7.57E-24 |
| **LQ** | HQ | 111 | 3 | X | 0.011 | 0.008 | 1.97E-02 |
| **LQ** | HQ | 938 | 4 | 2L | 0.058 | 0.010 | 2.37E-93 |
| **LQ** | HQ | 278 | 4 | 2R | 0.048 | 0.008 | 8.68E-15 |
| **LQ** | HQ | 372 | 4 | 3L | -0.001 | 0.006 | 1.22E-01 |
| **LQ** | HQ | 504 | 4 | 3R | 0.007 | 0.003 | 6.14E-02 |
| **LQ** | HQ | 111 | 4 | X | 0.027 | 0.010 | 1.94E-06 |
| **LQ** | HQ | 938 | 5 | 2L | 0.026 | 0.001 | 1.39E-51 |
| **LQ** | HQ | 278 | 5 | 2R | 0.021 | 0.003 | 1.81E-12 |
| **LQ** | HQ | 372 | 5 | 3L | 0.023 | 0.001 | 1.53E-18 |
| **LQ** | HQ | 504 | 5 | 3R | 0.025 | 0.001 | 1.82E-36 |
| **LQ** | HQ | 111 | 5 | X | 0.014 | 0.000 | 4.43E-12 |
| **LQ** | HQ | 938 | 6 | 2L | 0.028 | 0.007 | 1.11E-35 |
| **LQ** | HQ | 278 | 6 | 2R | 0.075 | 0.001 | 3.15E-55 |
| **LQ** | HQ | 372 | 6 | 3L | 0.025 | 0.002 | 1.12E-19 |
| **LQ** | HQ | 504 | 6 | 3R | 0.034 | 0.006 | 1.93E-23 |
| **LQ** | HQ | 111 | 6 | X | 0.013 | 0.004 | 1.38E-01 |
| **HQ** | LQ | 14925 | 1 | 2L | 0.010 | 0.000 | 2.07E-239 |
| **HQ** | LQ | 4374 | 1 | 2R | 0.017 | 0.000 | 1.35E-130 |
| **HQ** | LQ | 4871 | 1 | 3L | 0.018 | 0.002 | 4.61E-100 |
| **HQ** | LQ | 3660 | 1 | 3R | 0.009 | 0.001 | 1.65E-16 |
| **HQ** | LQ | 1473 | 1 | X | 0.018 | 0.003 | 1.87E-44 |
| **HQ** | LQ | 14925 | 2 | 2L | -0.001 | 0.001 | 9.83E-01 |
| **HQ** | LQ | 4374 | 2 | 2R | -0.002 | 0.002 | 9.87E-01 |
| **HQ** | LQ | 4871 | 2 | 3L | 0.009 | 0.003 | 4.81E-01 |
| **HQ** | LQ | 3660 | 2 | 3R | 0.041 | 0.004 | 9.46E-303 |
| **HQ** | LQ | 1473 | 2 | X | 0.003 | 0.003 | 1.62E-01 |
| **HQ** | LQ | 14925 | 3 | 2L | 0.051 | 0.006 | 0.00E+00 |
| **HQ** | LQ | 4374 | 3 | 2R | 0.050 | 0.005 | 0.00E+00 |
| **HQ** | LQ | 4871 | 3 | 3L | -0.007 | 0.004 | 7.80E-19 |
| **HQ** | LQ | 3660 | 3 | 3R | 0.008 | 0.001 | 5.30E-10 |
| **HQ** | LQ | 1473 | 3 | X | 0.013 | 0.005 | 1.64E-19 |
| **HQ** | LQ | 14925 | 4 | 2L | 0.082 | 0.003 | 0.00E+00 |
| **HQ** | LQ | 4374 | 4 | 2R | 0.054 | 0.001 | 0.00E+00 |
| **HQ** | LQ | 4871 | 4 | 3L | 0.031 | 0.002 | 1.23E-267 |
| **HQ** | LQ | 3660 | 4 | 3R | 0.023 | 0.004 | 1.35E-130 |
| **HQ** | LQ | 1473 | 4 | X | 0.006 | 0.004 | 4.48E-03 |
| **HQ** | LQ | 14925 | 5 | 2L | 0.022 | 0.005 | 0.00E+00 |
| **HQ** | LQ | 4374 | 5 | 2R | 0.024 | 0.002 | 8.22E-243 |
| **HQ** | LQ | 4871 | 5 | 3L | 0.026 | 0.002 | 0.00E+00 |
| **HQ** | LQ | 3660 | 5 | 3R | 0.039 | 0.002 | 0.00E+00 |
| **HQ** | LQ | 1473 | 5 | X | 0.020 | 0.003 | 1.31E-59 |
| **HQ** | LQ | 14925 | 6 | 2L | 0.021 | 0.004 | 0.00E+00 |
| **HQ** | LQ | 4374 | 6 | 2R | 0.009 | 0.001 | 5.95E-31 |
| **HQ** | LQ | 4871 | 6 | 3L | 0.016 | 0.000 | 3.92E-127 |
| **HQ** | LQ | 3660 | 6 | 3R | 0.019 | 0.003 | 3.97E-101 |
| **HQ** | LQ | 1473 | 6 | X | 0.015 | 0.001 | 2.91E-54 |

**Figure S10**. Pearson’s correlation coefficients were used to quantify the extent to which allele frequency movement between the HQ and LQ treatments were more correlated than expected by chance. (A) Correlation coefficients for all SNPs genome-wide and separately for SNPs on each chromosomal arm (red lines), relative to an empirical null distribution of correlations generated via N = 100 permutations of the empirical data. Observed correlations fall above the 99^th^ percentile of permuted values in all cases. (B/C) Correlations (red line) in allele frequency movement for the subset of SNPs exhibiting evidence of selection (GLM FDR < 0.05 and an allele frequency change > 2%) in (B) HQ (N = 29,303 total SNPs) and (C) LQ (N = 2,203 total SNPs). The grey distributions in (B) and (C) correspond to N = 100 random subsets of empirical data (A) of the same length as the focal correlation (red line).

**Table S8**. Pearson’s correlation coefficients were calculated for all assayed SNPs (All Sites) as well as the subset of sites displaying independent evidence of selection in the HQ and LQ treatments (see methods). Asterisks indicate that this value falls outside of the distributions generated by permutations of the allele frequency data, or with subsets of the SNPs with the same length and focal comparison.

| Genomic Region | All Sites | LQ Sites | HQ Sites |
| --- | --- | --- | --- |
| 2L | 0.58621* | 0.92962* | 0.87807* |
| 2R | 0.44739* | 0.89958* | 0.84393* |
| 3L | 0.28196* | 0.68690* | 0.65166* |
| 3R | 0.38686* | 0.81919* | 0.73747* |
| X | 0.19857* | 0.63991* | 0.70858* |
| Genome | 0.41877* | 0.86392* | 0.82338* |
